# Supplementary material for: Unveiling the Bioactive Potential of the Invasive Jellyfish Phyllorhiza punctata Through Integrative Transcriptomic and Proteomic Analyses
Source: Biomolecules. 2025 Aug 4;15(8):1121. doi: 10.3390/biom15081121 (PMC12383608; doi:10.3390/biom15081121)
Supplement: Supplementary file 1 [file biomolecules-15-01121-s001.zip › Suplementary Material/FileS3/PEPFOLD4/XP_065058610_662/clusters.html]

 PEP-FOLD clusters


Download archive of all models  here   

|  |  |  |
| --- | --- | --- |
| # | file | sOPEP |
| # cluster 1 - Size: 6 |  |  |
|  | XP\_065058610\_662-model1 | -11.409 |
|  | XP\_065058610\_662-model1.1 | -11.409 |
|  | XP\_065058610\_662-model1.2 | -11.409 |
|  | XP\_065058610\_662-model1.3 | -11.409 |
|  | XP\_065058610\_662-model1.4 | -11.409 |
|  | XP\_065058610\_662-model1.5 | -11.1889 |
| # cluster 2 - Size: 24 |  |  |
|  | XP\_065058610\_662-model2 | -5.85103 |
|  | XP\_065058610\_662-model2.1 | -5.7046 |
|  | XP\_065058610\_662-model2.2 | -5.47051 |
|  | XP\_065058610\_662-model2.3 | -5.47051 |
|  | XP\_065058610\_662-model2.4 | -5.47051 |
|  | XP\_065058610\_662-model2.5 | -5.47051 |
|  | XP\_065058610\_662-model2.6 | -5.47051 |
|  | XP\_065058610\_662-model2.7 | -5.47051 |
|  | XP\_065058610\_662-model2.8 | -5.42027 |
|  | XP\_065058610\_662-model2.9 | -5.42027 |
|  | XP\_065058610\_662-model2.10 | -5.40507 |
|  | XP\_065058610\_662-model2.11 | -5.40507 |
|  | XP\_065058610\_662-model2.12 | -5.39598 |
|  | XP\_065058610\_662-model2.13 | -5.39598 |
|  | XP\_065058610\_662-model2.14 | -5.39598 |
|  | XP\_065058610\_662-model2.15 | -5.32956 |
|  | XP\_065058610\_662-model2.16 | -5.32956 |
|  | XP\_065058610\_662-model2.17 | -5.32192 |
|  | XP\_065058610\_662-model2.18 | -5.30885 |
|  | XP\_065058610\_662-model2.19 | -5.24972 |
|  | XP\_065058610\_662-model2.20 | -5.21964 |
|  | XP\_065058610\_662-model2.21 | -5.18068 |
|  | XP\_065058610\_662-model2.22 | -5.17085 |
|  | XP\_065058610\_662-model2.23 | -5.11924 |
| # cluster 3 - Size: 64 |  |  |
|  | XP\_065058610\_662-model3 | -5.21801 |
|  | XP\_065058610\_662-model3.1 | -5.21801 |
|  | XP\_065058610\_662-model3.2 | -5.21801 |
|  | XP\_065058610\_662-model3.3 | -5.20708 |
|  | XP\_065058610\_662-model3.4 | -5.20708 |
|  | XP\_065058610\_662-model3.5 | -5.20708 |
|  | XP\_065058610\_662-model3.6 | -5.20708 |
|  | XP\_065058610\_662-model3.7 | -5.20076 |
|  | XP\_065058610\_662-model3.8 | -5.20076 |
|  | XP\_065058610\_662-model3.9 | -5.20076 |
|  | XP\_065058610\_662-model3.10 | -5.2003 |
|  | XP\_065058610\_662-model3.11 | -5.19338 |
|  | XP\_065058610\_662-model3.12 | -5.19338 |
|  | XP\_065058610\_662-model3.13 | -5.18466 |
|  | XP\_065058610\_662-model3.14 | -5.18466 |
|  | XP\_065058610\_662-model3.15 | -5.18459 |
|  | XP\_065058610\_662-model3.16 | -5.18459 |
|  | XP\_065058610\_662-model3.17 | -5.18092 |
|  | XP\_065058610\_662-model3.18 | -5.17882 |
|  | XP\_065058610\_662-model3.19 | -5.17882 |
|  | XP\_065058610\_662-model3.20 | -5.17604 |
|  | XP\_065058610\_662-model3.21 | -5.17604 |
|  | XP\_065058610\_662-model3.22 | -5.17555 |
|  | XP\_065058610\_662-model3.23 | -5.17153 |
|  | XP\_065058610\_662-model3.24 | -5.17153 |
|  | XP\_065058610\_662-model3.25 | -5.16368 |
|  | XP\_065058610\_662-model3.26 | -5.16299 |
|  | XP\_065058610\_662-model3.27 | -5.16068 |
|  | XP\_065058610\_662-model3.28 | -5.15702 |
|  | XP\_065058610\_662-model3.29 | -5.15702 |
|  | XP\_065058610\_662-model3.30 | -5.14759 |
|  | XP\_065058610\_662-model3.31 | -5.14522 |
|  | XP\_065058610\_662-model3.32 | -5.12861 |
|  | XP\_065058610\_662-model3.33 | -5.12835 |
|  | XP\_065058610\_662-model3.34 | -5.12835 |
|  | XP\_065058610\_662-model3.35 | -5.12641 |
|  | XP\_065058610\_662-model3.36 | -5.12641 |
|  | XP\_065058610\_662-model3.37 | -5.11913 |
|  | XP\_065058610\_662-model3.38 | -5.10578 |
|  | XP\_065058610\_662-model3.39 | -5.10578 |
|  | XP\_065058610\_662-model3.40 | -5.10359 |
|  | XP\_065058610\_662-model3.41 | -5.09867 |
|  | XP\_065058610\_662-model3.42 | -5.09074 |
|  | XP\_065058610\_662-model3.43 | -5.09067 |
|  | XP\_065058610\_662-model3.44 | -5.09005 |
|  | XP\_065058610\_662-model3.45 | -5.07505 |
|  | XP\_065058610\_662-model3.46 | -5.07332 |
|  | XP\_065058610\_662-model3.47 | -5.06813 |
|  | XP\_065058610\_662-model3.48 | -5.06311 |
|  | XP\_065058610\_662-model3.49 | -5.05253 |
|  | XP\_065058610\_662-model3.50 | -5.05253 |
|  | XP\_065058610\_662-model3.51 | -5.04983 |
|  | XP\_065058610\_662-model3.52 | -5.03882 |
|  | XP\_065058610\_662-model3.53 | -5.03727 |
|  | XP\_065058610\_662-model3.54 | -5.02715 |
|  | XP\_065058610\_662-model3.55 | -5.02193 |
|  | XP\_065058610\_662-model3.56 | -5.01981 |
|  | XP\_065058610\_662-model3.57 | -5.01898 |
|  | XP\_065058610\_662-model3.58 | -4.98013 |
|  | XP\_065058610\_662-model3.59 | -4.96028 |
|  | XP\_065058610\_662-model3.60 | -4.93932 |
|  | XP\_065058610\_662-model3.61 | -4.93702 |
|  | XP\_065058610\_662-model3.62 | -4.93602 |
|  | XP\_065058610\_662-model3.63 | -4.8416 |
| # cluster 4 - Size: 2 |  |  |
|  | XP\_065058610\_662-model4 | -4.991 |
|  | XP\_065058610\_662-model4.1 | -4.9144 |
| # cluster 5 - Size: 4 |  |  |
|  | XP\_065058610\_662-model5 | -4.94324 |
|  | XP\_065058610\_662-model5.1 | -4.89517 |
|  | XP\_065058610\_662-model5.2 | -4.83259 |
|  | XP\_065058610\_662-model5.3 | -4.83147 |
|  |  |  |
